# The views of stakeholders on controlled access schemes for high-cost antirheumatic biological medicines in Australia

**DOI:** 10.1186/1743-8462-4-26

**Published:** 2007-12-20

**Authors:** Christine Y Lu, Jan Ritchie, Ken Williams, Ric Day

**Affiliations:** 1 Department of Ambulatory Care and Prevention, Harvard Medical School, Boston, Massachusetts, USA; 2 School of Public Health and Community Medicine, University of New South Wales, Sydney, NSW, Australia; 3 School of Medical Sciences, University of New South Wales, Australia; 4 Department of Clinical Pharmacology and Toxicology, St Vincent's Hospital Sydney, Australia

## Abstract

**Background:**

In Australia, government-subsidised access to high-cost medicines is "targeted" to particular sub-sets of patients under the Pharmaceutical Benefits Scheme to achieve cost-effective use. In order to determine how this access system could be improved, the opinions of key stakeholders on access to biological agents for rheumatoid arthritis were explored.

**Methods:**

Thirty-six semi-structured interviews were conducted with persons from relevant stakeholder groups. These were transcribed verbatim, and analysed thematically.

**Results:**

Controlled access to expensive medicines was considered to be equitable and practical; however, there was disagreement as to the method of defining the target patient populations. Other concerns included timeliness of access, excessive bureaucracy, and the need for additional resources to facilitate the scheme. Collaboration between stakeholders was deemed important because it allows more equitable distribution of limited resources. The majority considered that stakeholder consultation should have been broader. Most wanted increased transparency of the decision-making process, ongoing and timely review of access criteria, and an increased provision of information for patients. More structured communication between stakeholders was proposed.

**Conclusion:**

The Pharmaceutical Benefit Scheme is adapting to meet the changing needs of patients. Provision of subsidised access to high-cost medicines in a manner that is affordable for individuals and society, and that is equitable and efficiently managed is challenging. The views of stakeholders on targeted access to anti-rheumatic biological medicines in Australia acknowledged this challenge and provided a number of suggestions for modifications. These could serve as a basis to inform the debate on how to change the processes and policies so as to improve the scheme.

## Introduction

Drug reimbursement systems grapple with the challenges of funding innovative but costly medicines in the face of increasing cost constraints, in part due to rapidly increasing demands for access to these drugs by consumers and prescribers. In Australia, government-subsidised access to prescription medicines is provided through the Pharmaceutical Benefits Scheme (PBS). Decisions on drug subsidy are based on assessment by the Pharmaceutical Benefits Advisory Committee (PBAC), which evaluates incremental effectiveness and cost-effectiveness of the medicine [1]. Criteria for access to PBS listed medicines are based on clinical trial evidence and economic evaluations.

Increasingly, there is collaboration between the PBAC, the sponsor, and medical organisations (e.g. Australian Rheumatology Association) to develop access criteria. A representative example of such a collaboration is the consultation process that contributed to the decision to subsidise and the criteria to access etanercept, a high-cost biological medicine for treating rheumatoid arthritis (RA). This process set a new paradigm for analogous PBS decisions [2,3]. A broad definition of "high-cost medicines" in Australia is medicines whose acquisition cost is greater than A$10,000 per patient per treatment course [4]. Although not directly involved in the consultation process, there was considerable support and lobbying activities by consumer representatives (namely the Arthritis Foundation of Australia) for 'targeted' access to high-cost biologicals. Access to high-cost medicines under the PBS is tightly regulated, requiring approval by Medicare Australia (a government body that administers the PBS) before it can be prescribed with subsidy by a doctor. Subsidised access to the anti-rheumatic biologicals is only provided for patients who meet criteria for both starting and continuing therapy. These criteria include both clinical and laboratory based measures (Table 1) [2]. Other biologicals subsequently subsidised by the PBS for treating RA are: infliximab, adalimumab, anakinra, and rituximab.

**Table 1:** Access arrangements for biological agents for RA under the PBS

	**Authority requirements**
**Criteria for initiating**	• Severe active disease:
**treatment**	a) elevated levels of inflammatory markers (ESR > 25 mm/hour or CRP > 15 mg/L)
	b) swollen and tender joints – a total of > 20 joints, or > 4 major joints (elbow, wrist, knee, ankle, shoulder, hip)
	• A record of rheumatoid factor positive status (this requirement was removed as of June 2005)
	• Failure to achieve adequate response to a step-up sequence of treatment with conventional DMARDs:
	a) monotherapy with methotrexate (20 mg per week)
	b) a combination of methotrexate (> 7.5 mg per week) and 2 other DMARDs for at least 3 months
	c) leflunomide, leflunomide with methotrexate, or cyclosporin for at least 3 months
	• Evidence of intolerance or contraindication to DMARDs
	• Patients required to sign a 'patient acknowledgement form'
	• Treatment is approved for 16 weeks only (treatment of 22 weeks is approved for infliximab)
**A patient agreement process**	• A **Patient Acknowledgement Form **to be signed by patients to acknowledge that PBS subsidised treatment will only continue if the predetermined response criteria are achieved at 12 weeks
**Criteria for continuing treatment**	• Clinical outcomes are evaluated according to predetermined quantifiable criteria at 12 weeks:
	a) Reduction in levels of inflammatory markers, ESR < 25 mg/hour, or CRP < 15 mg/L, or 20% from baseline levels
	b) Reduction in the total number of joint count by 50%
**'Interchangeability' (introduced December 2004)**	• Patients approved to commence PBS subsidised biological treatment are allowed to switch to an alternate biological agent at any time
**Restricted prescribing rights**	• Prescription only by specialist rheumatologists initially. Prescribing rights were extended to clinical immunologists with expertise in the management of RA as of February 2004
**'Risk-sharing' arrangement**	• Annual PBS expenditure for the tumour necrosis factor inhibitors group was predicted to be up to A$140 million
	• Expenditure above this figure to be covered by the sponsoring pharmaceutical companies (details not clear from public documents)

ESR = erythrocyte sedimentation rate

CRP = C-reactive protein

DMARDs = disease-modifying anti-rheumatic drugs

'Stakeholders', for the purpose of this study, were defined as individuals or groups of people having the potential to influence the decisions on the access arrangements, as well as those affected by the PBS restrictions. In reviewing the published literature, no studies apart from our own have examined the views of stakeholders regarding this novel approach to control subsidised access to high-cost medicines. This study examined the perceptions and experiences of stakeholders with respect to access to high-cost medicines under Australia's PBS using, as an example, the anti-rheumatic biologicals.

## Methods

In-depth semi-structured interviews were conducted with participants drawn from the stakeholder groups that were involved in access to biologicals through the PBS. Based on our definition of "stakeholders" in the case of anti-rheumatic biologicals, interviewees included were rheumatologists, patients with RA, government advisors, consumer advocates, public servants, and pharmaceutical company spokespersons. Interviewees were asked to declare any potential conflicts of interest, that is, any previous or current advisory role with a pharmaceutical company, or in any other committees or organisations that have a vested interest in the PBS listings.

Purposeful sampling of 'information-rich' individuals [5,6], especially those involved in formulating the criteria, was undertaken. This led to inclusion of additional interviewees i.e. the so-called 'snowball sampling technique' [5]. Interviews were conducted using a guide based on findings from the first phase of the study [7] by the same investigator (CL), a research pharmacist. The main questions put to interviewees are listed in Table 2. Each interview, lasting 45–80 minutes, was recorded and transcribed verbatim.

**Table 2:** Main interview questions

1. What are the sources of information you used before prescribing the drug, or during the course of the treatment?
2. What do you see as the primary objective of the PBS arrangements for access?
3. What do you see as the strengths and weaknesses of the access scheme?
4. Have you been involved in the consultation process in regards to developing restrictions or arrangements to access the TNF inhibitors via the PBS? If yes,
i. Can you briefly describe your role in the consultation process?
ii. Who else took part in the consultation process?
5. Who do you think should take part in the consultation process for formulating the arrangements or restrictions for access?
6. What is the extent of your contact with other rheumatologists, local general practitioners, administrators, consumer organization, the PBAC? And what were the purposes of these contacts?
7. What do you see as the role and responsibility of the prescribers? The PBAC? The industry? Arthritis Foundation?
8. Who, in your view, has responsibility in informing/'educating' the prescribers (and the public) regarding PBS restriction changes, or new complex PBS restrictions?
9. How important an advance do you think the consultation approach and access arrangements represent?
10. If a new and expensive drug comes along that is a significant advance for the treatment of a chronic disease, what differences would you like to see in the process of getting it listed and using it?

NVivo 2.0 software was used to manage the data and to assist the coding for major concepts arising inductively from the data. Three investigator independently categorised these concepts thematically before meeting to reach consensus on theme choice [8,9].

A variety of techniques was used to confirm the methodological rigour. These included verifying the meaning ascribed to interviewees by offering them the opportunity to review edited transcripts [9], using researcher triangulation to arrive at consensus on interpretation of the data [9], using triangulation of multiple data sources (namely, individuals from different stakeholder groups) to counterbalance weaknesses of one source by strengths in another [9], searching for negative or discrepant cases to enhance interrogation of the data, and continuing to add interviews until overall data saturation was achieved [10].

The study was approved by the Human Research Ethics Committees of St Vincent's Hospital Sydney, and the University of New South Wales, Australia.

## Results

Thirty-six interviews were conducted between 2004–2005 (Table 3). None of the government advisors, public servants, consumer representatives, or patients had held advisory positions or roles on behalf of a pharmaceutical company. Four of the eight rheumatologists had been or were on at least one advisory committee of a pharmaceutical company. Marked differences were not found in the views of rheumatologists who had held an advisory position with a pharmaceutical company versus those who had not. Most rheumatologists (7 out of 8) had been in practice for more than 10 years; the number of patients each rheumatologist treated with biologicals under the PBS ranged from 2 to 16. Most patients (5 out of 6) were prescribed etanercept. One nurse was included in the study opportunistically.

**Table 3:** Characteristics of interview participants (n = 36)

		**Number of participants**
**Age group**		
	18–29 years	1
	30–39 years	5
	40–49 years	8
	50–59 years	14
	60 years and over	8

**Sex**		
	Male	13
	Female	23

**Stakeholder group**		
	Rheumatologist	8
	Patient	6
	Government advisor	5
	Public servant	8
	Consumer representative	5
	Pharmaceutical industry representative	3
	Clinical nurse	1

Five major themes emerged: *resource rationing*, *excessive bureaucracy*, *partnerships and inclusive decision-making*, *education*, and *review*. All were related to the perceived fairness of the process and were essential determinants of support for the access criteria by stakeholders.

### 1. Resource rationing

Providing access to expensive medicines through a publicly-funded system (the PBS) was seen by all participants as equitable and a feasible approach. Targeting access was supported as a necessary form of "resource rationing". The vast majority acknowledged that there were finite resources available for the possible range of diseases. Targeting access to sub-groups of patients who most need treatment and would gain most benefit was in keeping with spending "for value". However, interviewees disagreed about how the appropriate target patient population should be defined. A government advisor recognised the tensions between system-wide and individual needs:

"It [controlled access] is entirely defensible in terms of a population utility concept. It's very difficult at an individual patient level."

Some participants suggested broader, initial access. The risk of excessive uptake of the biologicals by patients outside the PBS criteria was considered, by these participants, to be limited given the other controls that were applied, namely, risk-sharing between sponsors and government via *price-volume *agreements, and the *continuation rule *that limited ongoing access to those who responded to treatment. All agreed that the criteria should be based on sound clinical evidence. However, some criteria were seen as "arbitrary" and "potentially unfair" particularly when they were "unsupported by published literature". A rheumatologist gave his opinion:

"I think ... the restriction to seropositive patients is unfortunate. Secondly, the reliance on laboratory indices is a concern ... "

The requirement for 'positive rheumatoid factor' (seropositive) was removed shortly after the completion of this study and such opinions may have been rescinded subsequently.

The requirement to first trial existing, less costly therapies was considered to be reasonable. Limiting prescribing rights to sub-specialist physicians (rheumatologists, immunologists) was deemed safe and appropriate, given the expense of the medicines, the severity of RA in the affected patient population, and long-term safety concerns. A patient voiced the predominant view:

"I think it's good that people have to have tried all the other cheaper medicines, that's fair enough. Why should the government pay for a really expensive medicine when methotrexate works and also, we don't know yet what the long-term side effects will be."

While monitoring patient outcomes was supported, patients were apprehensive that an effective treatment might be withdrawn if one missed the threshold response to qualify for ongoing treatment. Further, prescribers might feel pressured to 'modulate' measurements of disease activity to benefit individual patients while, in effect, denying treatment for others. Some interviewees suggested that the *continuation rule *be removed, and others proposed increased flexibility with respect to the timing of the assessment, including less frequent assessment. A representative quote from one rheumatologist illustrates this view:

"I think a system that had more flexibility for the renewal, possibly by making the reapplication less frequent, or having a larger window within which the appropriate blood test could be acquired, or allowing so-called unacceptably high ESR [erythrocyte-sedimentation-rate] or CRP [C-reactive-protein] or joint count for a period of time, until things settle down again... a little less anxiety-provoking for the patients would be good."

However, government advisors and public servants believed that the interpretation of joint tenderness and swelling by clinicians and patients, being subjective, allowed some flexibility. Further, they believed that assessment of eligibility was flexible because applications for subsidised treatment were assessed by pharmacists and medical advisors from Medicare Australia, the responsible agency. Both prescribers and patients believed that clinicians who see individual patients should have such discretion because, as a patient put it: "people in government aren't educated about the disease [RA]". A government advisor voiced the opposing view about allowing flexibility with borderline cases:

"the problem with the high-cost drugs is it's very difficult to be flexible because small changes in flexibility start to blow budgets out to massive amounts of money and that means that the whole principle of equity and equitable access to these things are not obeyed. ... I don't think the uncertainty can be borne only by Australian taxpayers."

The *Patient Acknowledgement Form *was seen as a contract between the patient and the government, and reduced the direct pressure on the individual rheumatologists. Some rheumatologists and patients saw this agreement as "pointless" because treatment would be discontinued even if the patients disagreed, and it was unlikely that patients would wish to continue if the treatment was ineffective. A common view of patients was: "when you're desperate you'll sign anything."

### 2. Excessive Bureaucracy

The access scheme controlled the usage and expenditure of biologicals effectively in the first years of operation. This was seen as a good outcome by the majority. However, there was concern by some that the use of biologics was inappropriately low.

Most had the view that medical care had become "more bureaucratic" as a result of the PBS processes. A rheumatologist commented:

"It seems to be a very bureaucratic way of giving medical care, rather than a normal doctor-patient relationship... it's eliminating the ability of the doctor to show some level of discretion..."

The majority thought the application process was administratively burdensome. Patients were anxious about coordination of laboratory tests, joint assessment, application forms, and ordering the medicine from pharmacies in order to obtain ongoing supply. Physicians experienced difficulties in locating documents including records of laboratory tests and details of treatment history, some of which depended upon other clinicians who had treated patients previously. However, improved documentation was seen as a good outcome overall, and an application for access was easier for more recently diagnosed patients.

Prescribers were uneasy that there was no recompense for the substantial additional time to undertake the tasks to apply for access for their patients. Assistance (e.g. from nurses) was in general unavailable as the majority of rheumatologists in Australia work in private practice. Where nurses were available, considerable workload was imposed on them. A nurse was concerned about the impact on hospital resources:

"In a lot of places I think that nurses are doing a lot of the work. And there's nowhere that this has been taken into consideration with staff and issues and so on. It's added at least eight hours a week to my workload. ...."

There was also an increase in the use of other resources. A public servant noted that greater resources were needed to administer this complex scheme:

"We've got pharmacists on call and they are paid more than admin staff would be paid. We have to go through a more intensive process in order to approve the application because we lose more money if we approve it wrongly."

The requirement that there be an adequate trial of conventional anti-rheumatic drugs promoted re-evaluation of previous treatments and a more comprehensive and aggressive use of anti-rheumatic drugs. Patients were more easily convinced to go through these steps with the 'incentive' that they might access the 'new' biologicals. A rheumatologist voiced the predominant view:

"It's forced our rheumatologists to re-look at the treatment that patients have already received ... in reassessing the patient and aiming to meet the PBS criteria, they actually get their rheumatoid arthritis under control before requiring a biological..."

The majority considered that this effect was beneficial and potentially systematised the treatment of RA nationwide.

### 3. Partnerships and inclusive decision-making

There was uniform support for the stakeholder collaboration and increased communication that contributed to the decision to subsidise biologicals. However, while government advisors and public servants commented that the representative rheumatologists were cooperative, the companies felt that the representative rheumatologists were less willing to collaborate. The consumers felt that communication with the Rheumatology Association and the government was insufficient. Further, some interviewees suggested that consultation among rheumatologists should be wider. An industry spokesperson commented on the negotiation skills of the representing rheumatologists:

"I don't think they [rheumatologists] had the skills, which is built up over experience, to be able to handle negotiations with government. People like 'gastros' have had good experience over quite a long time with new therapies, and oncologists and cardiologists as well. Rheumatologists are babes in the wood when it comes to dealing with government."

Some believed that doctors would adequately represent the views of patients while the majority view was that patients should have a more direct role. A common view was that patient representatives would need to be carefully selected and well-informed. Interviewees had mixed opinions about when patients should be involved in the process, but that they should at least be involved in developing the Patient Acknowledgement Form. Some interviewees believed that negotiation between the three primary groups (PBAC, medical specialists, companies) was sufficient and would avoid difficulties and lengthy negotiations potentially associated with wider consultation.

An urgent need recognised by the majority was for open discussion to engage the broader community to work out the overarching principles of allocation and access to medicines. A government advisor voiced the predominant view:

"The population as a whole needs to have some debate about where they want to spend their money, they are the ones that are funding it ultimately..."

Greater transparency around deliberations between stakeholders and the rationale behind the selected criteria, currently constrained by confidentiality requirements, was considered essential to enhance understanding of the system and to enable the PBAC to better defend its decisions and garner broader support.

"We need greater transparency. It's [important] people understanding the system, taking some ownership of the system, involving them through transparency, through education, being prepared to speak to them. The PBAC should defend the decisions. It's all to do with dialogue, with the partnerships. This is not a them-and-us system, this is our system." (government advisor)

A comment by a rheumatologist summarised the two key elements for future processes:

"I would like to see a more collaborative and more open approach, because I think a lot of the negotiations that eventually resulted in this listing were, whether deliberately or accidentally, shrouded in some secrecy and I know from the PBAC perspective there's been some talk of deliberations being made more transparent. If we were doing this process again and if I had the power to change something, those are the things I would like to see: direct consumer representation and an open and transparent process that we could watch evolving."

### 4. Education

A common view of stakeholders was that the clinicians at Medicare Australia readily assist with queries regarding applications. However, the majority felt that better provision of information on the criteria and application procedures, particularly in the early stages of implementation, was important. In addition to avoiding confusion and increasing efficiency, it would enhance support for the access criteria.

Concerns were expressed about insufficient information for patients and inadequate clinical knowledge of the biologicals among health professionals in general. Clinicians emphasised the importance of providing information on adverse reactions, notably risks of infection. This was potentially a difficult task because some patients feared that treatment might be stopped if they reported infections. Most patients felt that quality-of-life outweighed other considerations including the potential risks of treatment.

The industry interviewees felt that education was a major responsibility of industry particularly when medicines are subject to complex criteria for access. It was agreed that company representatives had been helpful in providing information to clinicians. However, most interviewees expressed a lack of faith in the companies to supply appropriate materials for patients or the public. Concerns were also raised about fragmented and misleading messages delivered by the media.

Enhanced provision of information on the rationale for restricting access, how criteria were decided, and the distinction between "effectiveness" and "cost-effectiveness" of medicines was advocated. The *Patient Acknowledgement Form *represented an opportunity to increase patient understanding that there were responsibilities and risks to be shared by all parties. Most thought that information provided by the Australian Rheumatology Association (clinical information on the medicines) and the National Prescribing Service (information about the PBS and its decisions) would be most trustworthy. Consumer organisations were identified as having a role in disseminating information and providing consumer support. Interviewees suggested that general practitioners and other health professionals, such as pharmacists, could be more involved in educating patients.

### 5. Review

Regular review of access criteria was recommended. Drug utilisation patterns and feedback from stakeholders should be analysed to refine the access arrangements. The risk-sharing agreement should also be reviewed. The processes of PBAC decision-making and stakeholder consultation would also benefit from regular evaluation. A public servant also proposed examining the administrative expenses associated with such a scheme:

"The administration costs [of Medicare Australia] are not a consideration for the PBAC. We need to do some cost-benefit analysis around the cost of building the appropriate systems [of access] and the benefit in reduction of use [of medicines] on PBS."

Some were disappointed that a prospective, formal evaluation of the access scheme had not been established. This was partly because stakeholders could not reach consensus on who should be responsible or what were appropriate funding sources. Australian rheumatologists had implemented independently a patient registry to track patient outcomes. Some interviewees suggested that government and the pharmaceutical industry should fund such a registry collaboratively. The fact that a comprehensive information system enabling evaluation had not been developed was a concern for most interviewees.

Some rheumatologists and patients considered it a weakness that a review panel for contentious cases had not been established, while others felt that, to a degree, special consideration had occurred informally via interactions between Medicare Australia and rheumatologists. A patient voiced frustration about the delayed review of the PBS criteria:

"I would like a true appeal system. I would like whoever's in power to try and rectify the situation quickly and that when they put in future criteria for any medicine, that they are very, very careful about the criteria."

## Discussion

By including individuals who represented the range of stakeholder groups with their different and potentially competing interests, this study sought to present a well-rounded picture of the access scheme. In-depth insights into stakeholders' views provide a basis to inform the broader debate on how subsidy systems for high-cost medicines can be improved. The major consensus finding was that while the the current access scheme for anti-rheumatic biologicals can be viewed as successful, stakeholder communication and involvement needs to be increased. A limitation of the study was that insights into some managerial perspectives were not obtained because administrators who assessed patient applications for access declined to participate due to concerns about privacy.

The innovations introduced to establish the access scheme were considered concordant with the expectation that government is responsible for providing access to effective medicines while balancing the need to use public resources wisely. Limiting access to sub-sets of patients, where need has been established and where use was cost-effective, was viewed as practical and equitable. The stakeholders were supportive of the proposition that it is possible to make high-cost medicines available and affordable for the community and individual patients. That this had been achieved with these drugs was accepted as an important accomplishment. However, timeliness of access to innovative medicines via the PBS in Australia, in comparison to comparable countries, was a concern. This was partly due to the registration process which often occurred later in Australia than in the USA or Europe. A definition of "timeliness of access to medicines" by interviewees of the six stakeholder groups was not actively explored in this study; an important issue that warrants further investigation.

Criteria for access were seen to be potentially 'unfair' when the evidence was not publicly available. For example, the requirement for *rheumatoid-factor-positive-status *to gain access was contentious at the time of the interviews. Most published pivotal studies did not exclude rheumatoid-factor-negative patients or analyse them as a subgroup. There was insufficient evidence in the public domain to support the view that rheumatoid-factor-positive-status was a factor predicting a better response to treatment with etanercept [11]. However, efficacy in rheumatoid-factor-negative patients had not been clearly established because of the small number of such patients in these trials [12]. This requirement for rheumatoid factor positivity has subsequently been removed. Sustainability of a subsidy system such as the PBS is dependent upon a rigorous and consistent process of drug review and increased transparency around the rationale underpinning decisions [13]. How the PBAC arrives at its recommendations for PBS listing was significantly limited by the "commercial-in-confidence" restrictions at the time of etanercept listing. An important milestone in this respect is that summaries of PBAC decisions have become available publicly recently [3,14]. Increasing transparency also increases accountability of all parties for decisions and performance of the system. These moves towards transparency were supported by the participants.

Open dialogue and declaration of potential conflicts of interest are important to building trust [15]. Increased communication and collaboration between stakeholders have been crucial steps that were initiated during the effort to subsidise the anti-rheumatic biologicals. However, the consumer participants identified a need for increasing the voice of patients and the public in order to enhance the quality of decisions, and acceptance of access criteria, a position supported by the majority of participants.

Increasing bureaucratic requirements are a threat to the acceptance and efficient running of such access systems. The administrative burden imposed on prescribers can be a barrier to enrolling deserving patients and a source of increased costs [16]. Most stakeholders, with the exception of the government advisors and public servants, proposed that more reliance on physicians' integrity to comply with PBS criteria should be considered an appropriate approach and would benefit patients.

A significant gap in knowledge was identified by health professionals (namely, general practitioners, pharmacists and nurses) and the community about the PBS and the increasing need to control access to medicines, in particular, high-cost medicines. General practitioners and pharmacists were identified as potentially important contributors to the success of targeted access schemes. They have an important role to play in educating patients about the use of medicines in chronic diseases such as RA and the PBS system in general. Adequate provision of information is critical to managing patient expectations and empowering them to participate in decision-making processes [17].

## Conclusion

The limited resources must be carefully used to provide needed, effective and safe medicines that are affordable for the individual and society in order to achieve optimal outcomes. Policy makers dealing with subsidised access to high-cost medicines might focus upon: increasing stakeholder involvement in decisions; better methods of defining target populations; improving the timeliness of access; increasing the flexibility for clinicians to make decisions while reducing bureaucratic red-tape; enhancing resources to administer systems of access; and improving communication and information based on increased transparency.

## Competing interests

CL and JR have nothing to declare. KW has been a member of the Advisory Board to the sponsor for adalimumab. RD has been a member of the Advisory Board to sponsors for adalimumab, infliximab, and anakinra in Australia. KW and RD have also been contracted to undertake clinical trials of etanercept, infliximab, adalimumab, and anakinra. Recompense for these activities is placed in audited hospital trust funds for use in the research activities of the Clinical Pharmacology Department, St Vincent's Hospital, Sydney.

## Authors' contributions

CL, KW, and RD have made substantial contributions to the conception and design of the study, analysis and interpretation of the data, and drafting and revising the manuscript. JR has been involved in advising on the analysis, drafting the manuscript and revising it critically for intellectual content. All authors read and approved the final manuscript.

## References

[B1] Commonwealth Department of Health and Aged Care (2000). The Australian Health Care System, An Outline.

[B2] Lu CY, Williams KM, Day RO, March LM, Sansom L, Bertouch J (2004). Access to high cost drugs in Australia: Risk sharing scheme may set a new paradigm. BMJ.

[B3] Sansom L (2004). The subsidy of pharmaceuticals in Australia: processes and challenges. Aust Health Rev.

[B4] High Cost Drugs Working Party Drug and Therapeutics Committee Template for Formulary Submissions - Decision algorithm; NSW Therapeutic Advisory Group (cited 12 Oct 2006). http://www.ciap.health.nsw.gov.au/nswtag/high_cost_drugs-wp.html.

[B5] Patton MQ (2002). Qualitative research and evaluation methods.

[B6] Pope C, van Royen P, Baker R (2002). Qualitative methods in research on healthcare quality. Quality and Safety in Health Care.

[B7] Lu CY, Ritchie J, Williams KM, Day RO (2005). Recent developments in targeting access to high cost medicines in Australia.. Aus NZ Health Policy.

[B8] Pope C, Ziebland S, Mays N (2000). Qualitative research in health care: Analysing qualitative data. BMJ.

[B9] Patton MQ (1999). Enhancing the quality and credibility of qualitative analysis. Health Serv Res.

[B10] Strauss A, Corbin J (1998). Basics of qualitative research.

[B11] Lu CY, Williams KM, March LM, Bertouch J, Day RO (2004). Subsidised access to TNFa inhibitors: is the rationale for exclusion of rheumatoid factor negative patients defensible?. Med J Aust.

[B12] Sansom L (2004). Subsidised access to TNFa inhibitors: is the rationale for exclusion of rheumatoid factor negative patients defensible? [comment]. Med J Aust.

[B13] Morgan SG, McMahon M, Mitton C, Roughead E, Kanavos P, Menon D (2006). Centralized drug review processes in Australia, Canada, New Zealand, and the United Kingdom. Health Aff (Millwood).

[B14] Australian Government Department of Health and Ageing Pharmaceutical Benefits Scheme - Public Summary Documents. http://www.health.gov.au/internet/wcms/publishing.nsf/Content/public-summary-documents.

[B15] Smith R (2004). Transparency: a modern essential. BMJ.

[B16] Burton SL, Randel L, Titlow K, Emanuel EJ (2001). The ethics of pharmaceutical benefit management. Health Aff (Millwood).

[B17] Gibson JL, Martin DK, Singer PA (2005). Priority setting in hospitals: fairness, inclusiveness, and the problem of institutional power differences. Soc Sci Med.

